# Transcatheter Closure of Postinfarction Ventricular Septal Defect: A Case Report and Review of Literature

**DOI:** 10.15171/jcvtr.2015.17

**Published:** 2015

**Authors:** Mahmood M. Shabestari, Fereshteh Ghaderi, Ali Hamedanchi

**Affiliations:** ^1^ Atherosclerosis Prevention Research Center, School of Medicine, Mashhad University of Medical Sciences, Mashhad, Iran; ^2^ Atherosclerosis Prevention Research Center, Imam Reza Hospital, School of Medicine, Mashhad University of Medical Sciences, Mashhad, Iran; ^3^ Department of Cardiology and Intensive Care Medicine, University of Jena, Germany

**Keywords:** Myocardial Infarction, Ventricular Septal Rupture, Interventional Closure, Amplatzer Occluder Device

## Abstract

Ventricular septal rupture (VSR) is an uncommon but serious complication of acute myocardial infarction (MI), associated with a high mortality rate. Although early surgical treatment improves the prognosis, hospital mortality after emergency surgery remains high. Transcatheter closure of postmyocardial infarction ventricular septal defect (PIVSD) has emerged as a potential strategy in selected cases. Current interventional reports are mainly restricted to PIVSD closure in the chronic and subacute setting, which only give a short term result. Herein, we report a case of acute post-MI VSR that was successfully closed using an Amplatzer postinfarction muscular ventricular septal defect (PIMVSD) occluder device with good immediate and long-term outcomes. The patient had undergone urgent coronary artery bypass surgery 3 days earlier in the setting of acute MI.

## Introduction

Ventricular septal defect (VSD) complicated acute MI is an infrequent but catastrophic event with a grim prognosis. It occurs as a bimodal presentation with a higher incidence in the first 24 hours and then again 3 to 5 days after an acute MI.^1^ In postmortem research, VSRs have been classified into simple versus complex ruptures. Simple VSRs are straight, horizontal septal canals while complex VSRs travel serpiginously through the septum before exiting at a different level.^2,3^

When VSR does occur, clinical presentation is often ominous, commonly associated with extensive comorbidities, resulting in poor cardiac output, multiorgan failure, and death. Survival past one month without intervention is 6%.^3-5^ To date, the American College of Cardiology and American Heart Association (ACC/AHA) still advise immediate surgical closure of the VSR. However, high mortality rates are not unexpected with advanced patient age, and other factors including comorbidities, hemodynamic instability, and technical challenges of the surgical procedure. Therefore, many surgeons prefer to delay surgical VSD repair at least by two weeks to allow initial healing, firmer anchoring of suture and better support for patch material.^6,7^ This introduces a significant selection bias into surgical series, artificially inflating survival rates.^7^ An interventional approach is a less invasive option and might allow for immediate complete VSD closure or initial haemodynamic stabilization.

Herein, we report a case of a 73-year-old male with acute VSR, successfully closed by a percutaneous interventional technique with an excellent long term prognosis.

## Case Report

**Figure 1:**
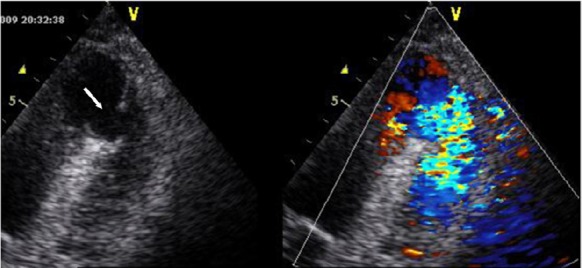
A close-up of the ventricular septum in an apical fourchamber view demonstrates 2D defect (white arrow) and turbulent systolic color Doppler flow across VSD before the procedure.

A 73-year-old man with a history of hypertension and dyslipidemia was admitted to our Cardiology Department due to anterior S-T segment elevation acute MI. He was hemodynamically stable and was initially treated with thrombolytic therapy. Urgent coronary angiography was planned because of the absence of reperfusion and hemodynamic deterioration. Transthoracic echocardiography revealed LV enlargement, akinesia of anterior, septal and all apical segments with left ventricular ejection fraction of about 25%. Mild to moderate MR was also noted. There was no post MI mechanical complication. An intra-aortic balloon pump was inserted and coronary angiography was performed which showed severe three-vessel disease that was not amenable to percutaneous intervention. Surgical revascularization was conducted on the second day of admission. After the operation, he was stable for three days, however, he subsequently developed progressive dyspnea, tachycardia and hypotension. Cardiovascular examination was remarkable for a new harsh holosystolic murmur heard maximally at the left lower sternal border. His blood pressure was 95/50 mm Hg and rales were present in the bases of the lungs. Transthoracic echocardiogram showed an apical moderate size simple VSD measuring by color Doppler echocardiography (LV side: 12 mm, RV side: 9 mm, apical rim: 8 mm) with bidirectional shunting (Figure 1). RV systolic pressure was estimated as 55 mm Hg from a tricuspid regurgitant jet velocity of 3.4 m/s. Due to the patient’s clinical condition, and his refusal to redo cardiac surgery, interventional VSD closure was planned under fluoroscopy and real time transesophageal echocardiography (TEE) (Vivid 3, GE, USA) guidance.

**Figure 2:**
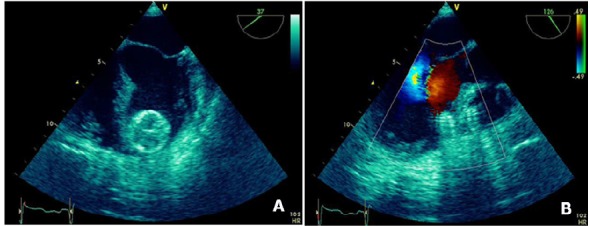
Transesophageal monitoring of ventricular septal defect closure: The LV disc of device has been deployed (A); Complete expansion of device with no residual shunt (B).

In this patient, because of difficulties in the routine arterial approach, we decided to advance the A_1_ catheter from the femoral vein access to RV, then to LV via VSD. A 0.035 inches ×3 m Amplatz guidewire was advanced to RV crossing VSD into LV, ascending and then descending aorta for better support. The delivery sheath was then advanced from the venous side to the LV cavity over the wire and the dilator and the wire was carefully removed. A 20-mm Amplatzer PIMVSD device (AGA Medical) was used. The occluder device was then delivered to the LV. The device was extruded from the sheath until the LV disc was opened under echocardiographic guidance to ensure that the device did not open in the mitral chordal apparatus (Figure 2A). It was then withdrawn toward the interventricular septum. After further satisfactory echocardiographic evaluation of septal alignment, the RV disc was also deployed and the device was released from the delivery cable (Figure 2B).

**Figure 3:**
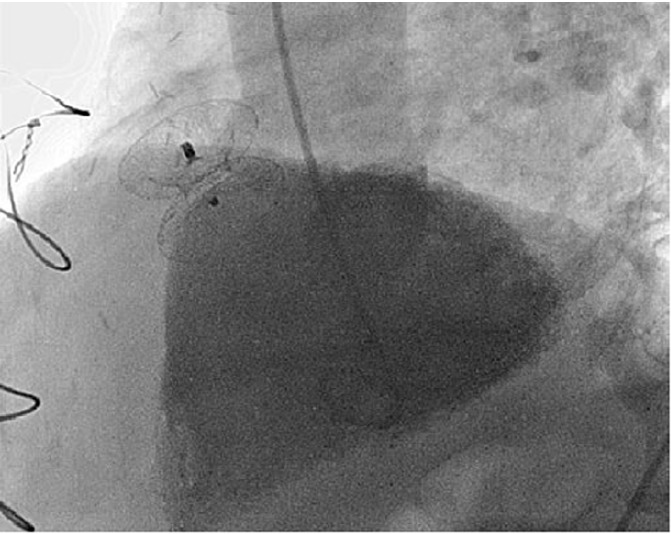
Left ventriculography in lateral view confirming good device position with no residual shunt.

Both TEE (Figure 2B) and left ventriculography (Figure 3) confirmed good placement of the device with no detectable residual shunt, demonstrating good device size selection. The patient showed immediate significant symptomatic improvement. He was transferred to the coronary care unit, and discharged seven days later. As of the date of this case report, the patient is alive and feeling well with no residual shunt detected by transthoracic echocardiography. No procedure-related complications have been recorded during these six years.

## Discussion

VSR occurs in 0.2% of MI cases, usually as early as 24 hours or as late as 5-6 days with a range of 1-14 days postinfarction period.^4^ Mortality is high, exceeding 90% in patients who go untreated. Acute VSR requires emergency operation regardless of the patient’s clinical status. However, the incidence rate of residual shunts following surgery is about 20% with a mortality rate close to 50%.^4-8^

Transcatheter closure of PMIVSD as a less invasive approach may improve survival rates in selected patients with suitable anatomy.^9^ The procedure is one of the most challenging in interventional cardiology because the margins of the defect may have necrotic borders and clinical condition of these patients is often poor. The first experience in 1998 by Landzberg and Lock involved percutaneous closure of Post-MI VSDs using older closure devices (Rashkind double umbrella).^3,8^ Based on the literature, a variety of devices have been used in this respect including atrial-septal-defect occluder (ASDo), muscular ventricular-septal-occluder (mVSDo) and recently a specific post-infarction VSD occluder developed by Amplatzer.^3,8^ It is not yet clear which occluder device is the best option for the treatment of a VSR. However, It seems that the use of ASDo is not an optimal treatment option, especially in the acute phase (within <7 days after VSR diagnosis). In Bialkowski et al study, interventional closure of PMIVSD cases was carried out mainly using atrial septal occluders. Procedure failure occurred in all patients with acute PMIVSD. The authors reported satisfactory results only in subacute and chronic phase cases.^10^

The PIMVSD Amplatzer devices show implantation success and short term results superior to the formerly used devices.^3,11^ The Amplatzer PIMVSD device is a nitinol construct with sizes ranging from 16 to 24 mm and a connecting waist of 10 mm in length. The LV and RV disks are 5 mm larger than the waist. In Holzer et al study (the US registry), an Amplatzer device was successfully implanted in 89% of the patients, although their 30-day mortality was still high (28%).^11^ In each of the reported cases in the US registry data the device chosen was based on having a waist at least 4 mm larger than the stretched diameter of the VSD found on TEE. In our case, we selected prosthesis 8 mm larger than the maximum diameter of the VSD. This oversizing may compensate for further enlargement of the defect caused by tissue necrosis. In such a situation, additional risk during the procedure may result from sending emboli of necrotic fragile tissue to the systemic or pulmonary circulation.^8^ Fortunately, we did not observe such events in our patient. Another point of concern is possible entrapment of the device in the mitral valve apparatus or damage to the tricuspid leaflets.^8,12^ Continuous TEE guidance during the procedure is strongly recommended to prevent such complications.^13^ Echocardiographic surveillance should not only monitor accurate deployment of devices, but also the anatomical integrity of surrounding structures both during and after the procedure.^12^

In another study by Calvert et al, percutaneous PMIVSD closure was attempted in 53 patients from 11 centers (the UK experience). Time duration from myocardial infarction to closure procedure was 13 days (first and third quartiles, five to 54). This series of unselected cases undergoing percutaneous VSD repair showed that the overall outlook remains poor for such high risk patients. However, those who survived and were discharged from the hospital had a good long-term prognosis, as observed in our patient. Long-term follow-up for patients who survived to hospital discharge was 395 days (first and third quartiles, 63–1522).^7^

To the authors’ knowledge, this case represents the longest term follow-up to date of a successful transcatheter closure of acute post-MI VSD with excellent outcome.

## Conclusion

Postinfarction VSDs are still associated with a very high morbidity and mortality. Transcatheter closure of PIVSD is a challenging but viable option for these critically unwell patients. We presented a case of acute post-MI VSR rejected for high risk repeat surgery in which percutaneous device closure was performed. The patient’s condition has been uneventful during the six-year follow up. Transcatheter closure in certain cases of acute post-MI VSR, with simple morphology, optimal apical rim and an acceptable size defect(<1.5 cm), may offer a promising alternative method to high risk surgery in these patients.

## Acknowledgments

The authors thank Dr. Ali Eshraghi and Dr. Toktam Moghiman for their contributions to the writing of this report.

## Ethical Issues

The study was approval by our local Ethics Committee.

## Competing Interests

Authors declare no conflict of interest in this study.
